# Supplementary material for: Differences in Carbon and Nitrogen Migration and Transformation Driven by Cyanobacteria and Macrophyte Activities in Taihu Lake
Source: Int J Environ Res Public Health. 2022 Dec 26;20(1):371. doi: 10.3390/ijerph20010371 (PMC9819403; doi:10.3390/ijerph20010371)
Supplement: Supplementary file 1 [file ijerph-20-00371-s001.zip › ijerph-2111797-supplementary.pdf]

Supplementary data

Differences in Carbon and Nitrogen Migration and Transformation Driven by Cyanobacteria and Macrophyte Activities in Taihu Lake

Chaonan Han*, Hao Wu, Ningning Sun, Yu Tang, Yan Dai and Tianhao Dai

School of Civil Engineering, Nanjing Forestry University, Nanjing 210037

Corresponding author: hcn_125@njfu.edu.cn; Tel.: +86–13022593296

Contents of this file

Supplementary methods for the determination of N species in the sediments.

Table S1. Water quality parameters in Taihu Lake.

Table S2. Abundance and diversity indices for bacterial communities in the sediments of Taihu Lake.

Supplementary methods for the determination of N species in the sediments

Sediment total nitrogen (S-TN) was determined by referencing the method in Liu's study [26]. A total of 0.1 g of sediment was placed into a 50 ml centrifuge tube, to which 20 ml of alkaline potassium persulfate digestion solution (0.24 mol/L NaOH, 0.074 mol/L $K_2S_2O_8$) was added. After that, the samples were digested at 120 °C and 0.15 MPa for 1 hour before being centrifugated to obtain the supernatant. The supernatant was used to determine S-TN through UV spectrophotometry [24].

Labile inorganic nitrogen species in the sediments were determined by referencing Liu's method [26]. A total of 0.5 g of sediment was placed into a 50 ml centrifuge tube, to which 20 ml of potassium chloride solution (1 mol/L KCl) was added. After that, the samples were shaken in an oscillator for 2 hours before being centrifugated to obtain the supernatant. The supernatant was used to determine nitrite, nitrate and ammonia in the sediments (KCl-NO_2^-, KCl-NO_3^-, KCl-NH_4^+) using naphthalyl-ethylenediamine photometry, UV spectrophotometry and Nessler's reagent, respectively [24].

Table S1. Water quality parameters in Taihu Lake.

Site	pH	DO (mg/L)	Temperature (°C)	EC (μs/cm)
East of lake	7.8	9.4	30.5	355
Meiliang Bay	8.2	13.5	31.5	364

Table S2. Abundance and diversity indices for bacterial communities in the sediments of Taihu Lake.

Layer	Site	Shannon	Simpson	ACE	Chao	Coverage
Surface	East of lake	6.19	0.0055	2462	2441	0.984
sediment	Meiliang Bay	6.32	0.0057	2675	2690	0.981
Deep	East of lake	6.29	0.0054	2532	2552	0.986
sediment	Meiliang Bay	6.44	0.0043	2802	2842	0.984
